# Radiation therapy and anti-tumor immunity: exposing immunogenic mutations to the immune system

**DOI:** 10.1186/s13073-019-0653-7

**Published:** 2019-06-20

**Authors:** Claire Lhuillier, Nils-Petter Rudqvist, Olivier Elemento, Silvia C. Formenti, Sandra Demaria

**Affiliations:** 1 Department of Radiation Oncology, Weill Cornell Medicine, Stich Radiation Oncology Center, 525 East 68th Street, New York, NY 10065 USA; 2 Department of Physiology and Biophysics, Caryl and Israel Englander Institute for Precision Medicine, Weill Cornell Medicine, 413 East 69th Street, New York, NY 10021 USA; 3 Sandra and Edward Meyer Cancer Center, Weill Cornell Medicine, 1300 York Avenue, New York, NY 10065 USA; 4 Institute for Computational Biomedicine, Weill Cornell Medical College, 1305 York Avenue, New York, NY 10021 USA; 5 Department of Pathology and Laboratory Medicine, Weill Cornell Medicine, 1300 York Avenue, New York, NY 10065 USA

## Abstract

The expression of antigens that are recognized by self-reactive T cells is essential for immune-mediated tumor rejection by immune checkpoint blockade (ICB) therapy. Growing evidence suggests that mutation-associated neoantigens drive ICB responses in tumors with high mutational burden. In most patients, only a few of the mutations in the cancer exome that are predicted to be immunogenic are recognized by T cells. One factor that limits this recognition is the level of expression of the mutated gene product in cancer cells. Substantial preclinical data show that radiation can convert the irradiated tumor into a site for priming of tumor-specific T cells, that is, an in situ vaccine, and can induce responses in otherwise ICB-resistant tumors. Critical for radiation-elicited T-cell activation is the induction of viral mimicry, which is mediated by the accumulation of cytosolic DNA in the irradiated cells, with consequent activation of the cyclic GMP-AMP synthase (cGAS)/stimulator of interferon (IFN) genes (STING) pathway and downstream production of type I IFN and other pro-inflammatory cytokines. Recent data suggest that radiation can also enhance cancer cell antigenicity by upregulating the expression of a large number of genes that are involved in the response to DNA damage and cellular stress, thus potentially exposing immunogenic mutations to the immune system. Here, we discuss how the principles of antigen presentation favor the presentation of peptides that are derived from newly synthesized proteins in irradiated cells. These concepts support a model that incorporates the presence of immunogenic mutations in genes that are upregulated by radiation to predict which patients might benefit from treatment with combinations of radiotherapy and ICB.

## Background

T cells can recognize differentiation antigens and other non-mutated self-antigens that are overexpressed by cancer cells in the context of sufficient inflammatory signals, which result from the release of damage-associated molecular pattern (DAMP) molecules [1, 2]. Over the past few years, numerous studies have demonstrated that a high mutational load (that is, a high number of non-germline, non-synonymous mutations per exome) is generally associated with improved responses of cancer patients to immune checkpoint blockade (ICB) therapy [3–6]. When the genes that contain these somatic mutations are translated at sufficient levels into proteins that, once degraded by the proteasome, generate peptides that bind with high affinity to major histocompatibility complex class I (MHC-I) molecules, tumor neoantigens are generated.

Neoantigens are known to be often highly immunogenic and represent key targets for T cells [7]. Therefore, targeting the tumor mutanome for individualized vaccination constitutes a promising strategy for increasing the responses of patients treated with ICB. Recently, several phase-I clinical trials have demonstrated the feasibility of personalized neoantigen vaccination for treatment of melanoma and glioblastoma patients, with the induction of neoepitope-specific T cells that were able to kill autologous tumor cells [8–10].

Although increased tumor mutational load theoretically leads to the accumulation of neoantigens, only a subset of mutated peptides are presented on MHC-I molecules, and among them, only a small percentage generate T-cell responses. Predicting which somatic mutations are immunologically relevant remains a challenge. Despite the efforts deployed by many groups to understand the characteristics of a neoantigen that can induce a strong T-cell response, this knowledge remains far from complete [11, 12]. The development of improved prediction algorithms to identify neoepitopes that bind with high affinity to the product of each human MHC allele will enhance the identification of potentially immunogenic mutations. Such algorithms will be enhanced by our improved ability to identify MHC-bound peptides using mass spectrometry [13].

Focal radiotherapy (RT) has been used for more than a century to attain local tumor control. The DNA damage caused by RT mediates its cytocidal effects, but is also responsible for many of the pro-inflammatory effects of RT because DNA that gains access to the cytosol of cancer cells and myeloid cells within the irradiated tumor microenvironment acts as a powerful DAMP [14, 15]. In pre-clinical studies, RT has been demonstrated to induce the activation of T cells that are directed against model antigens introduced into cancer cells, such as ovalbumin, and against some endogenous tumor antigens [16–18]. There is some evidence that T-cell activation against some tumor antigens also occurs in patients [19]. However, RT by itself is seldom able to induce T-cell responses that mediate abscopal effects (that is, the regression of non-irradiated metastases; Box 1), as reflected by the extremely rare occurrence of such effects [20, 21]. Nevertheless, the ability of RT to promote the activation of anti-tumor T cells has become clinically relevant with the advent of ICB therapy, with examples in both mice and patients showing that RT can help to overcome resistance to ICB [22–25].

In this article, we briefly review key features pertaining to the regulation of antigen processing and presentation by MHC-I, which have been studied mostly in the context of viral infections. We then propose that treatments that elicit a stress response in cancer cells, such as radiotherapy and chemotherapy, modulate the tumor neoantigen landscape by inducing the expression of genes that encode immunogenic mutations. We discuss evidence that supports this concept in the context of ionizing radiation, where antigenic modulation together with pro-inflammatory effects regulate the synergy between focal RT and immunotherapy. We extend the discussion to consider the role of the MHC class II (MHC-II) pathway in presenting the cancer mutanome to CD4 T cells, and we describe additional types of tumor neoantigens that are emerging as targets of anti-tumor T cells, such as antigens generated by post-translational modifications (PTMs).

## The rules of antigen presentation by MHC-I molecules

In order to eliminate aberrant (that is, virally infected or transformed) cells, CD8 cytotoxic T cells must recognize antigens displayed by MHC-I molecules on the surface of the aberrant cells. MHC-I molecules, which are expressed by all nucleated cells in the body, have evolved to provide information to the immune system about internal changes in an individual cell that constitute a danger to the organism. The molecular mechanisms that regulate antigen presentation by MHC-I have been described in detail in several excellent reviews (for example, [26, 27]). We focus on the salient features of this process that are relevant for understanding how neoantigen presentation by cancer cells can be modulated by treatments, such as RT and genotoxic chemotherapy.

**Fig. 1:**
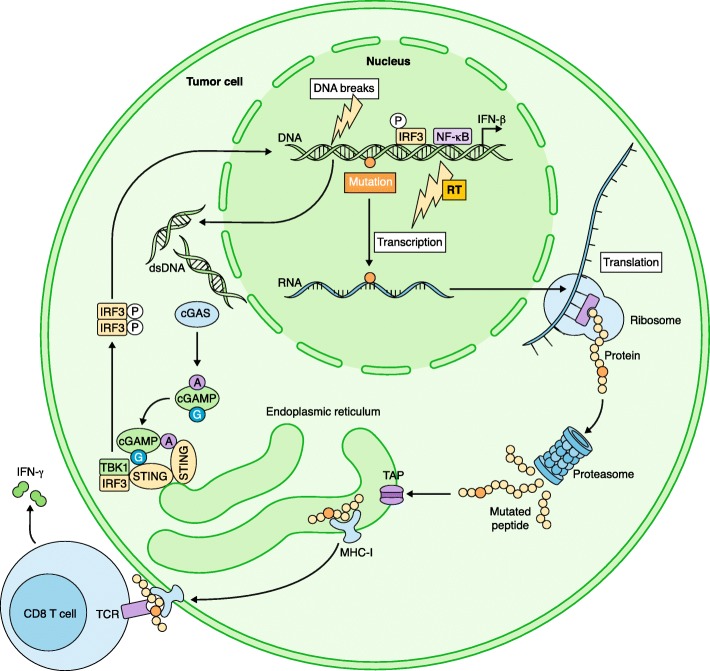
Radiation therapy can expose immunogenic mutations for MHC-I presentation on the surface of cancer cells. In response to DNA damage that is caused by radiation, double-stranded DNA (dsDNA) accumulates in the cytosol, where it triggers a cellular response similar to that induced by a viral infection. Cytosolic dsDNA binds to cyclic GMP-AMP synthase (cGAS), stimulating the production of cGAMP (cyclic guanosine monophosphate–adenosine monophosphate), which activates Stimulator of interferon genes (STING). Downstream of STING the type I interferon (IFN-I) and NF-κB pathways are activated, resulting in the production of IFN-β and other pro-inflammatory cytokines and in the induction of IFN-stimulated genes, including immunoproteasome subunits. The expression of multiple genes encoding proteins that are involved in DNA damage repair and cell-cycle regulation is also induced. These genes frequently contain mutations. After translation, the mutated proteins will be processed by the (immuno)proteasome and degraded into shorter peptides (8–11 amino acids long) that will enter the endoplasmic reticulum via the transporter associated with antigen processing (TAP) complex. Peptides that bind to MHC-I molecules with sufficient affinity will then be presented at the tumor cell surface, where they can be recognized by CD8 T cells. *RT* radiotherapy, *TCR* T-cell receptor

MHC-I molecules present peptides of 8–11 amino acids in length that are derived from the proteasomal degradation of intracellular proteins. These small peptides are translocated into the endoplasmic reticulum (ER) by the transporter associated with antigen processing (TAP) (Fig. 1). In the ER, the MHC-I components—a polymorphic heavy chain and a light chain called β2-microglobulin (β2m)—are partially folded and stabilized by different chaperone proteins [28]. Once a peptide binds to an MHC-I molecule, the chaperones are released and the peptide–MHC complex is transported via the Golgi complex to the plasma membrane.

Any peptide that binds with sufficient affinity to stabilize the complex of the MHC-I heavy chain with β2m can theoretically be presented. Therefore, a number of mechanisms have evolved to enable the distinction between self and non-self peptides. First, T cells that are reactive to a vast array of self-antigens are eliminated in the thymus, a process known as central tolerance; second, T-cell-intrinsic and -extrinsic regulatory mechanisms are in place to maintain peripheral tolerance [29]. Key to T-cell specificity is the requirement for two signals in order for the T cell to be activated. The T-cell receptor (TCR) provides the first signal upon binding to the MHC–antigen complex, and the co-receptor CD28 delivers the second signal upon binding to costimulatory molecules CD80 and CD86 [30]. The expression of costimulatory molecules is largely restricted to professional antigen-presenting cells such as dendritic cells (DCs), and only a special subset of DCs, known as conventional DC1, has the ability to take up antigen(s) from other infected or transformed cells and to cross-present them on MHC-I in order to activate CD8 T cells [29, 31]. In the absence of disease, scheduled cell death serves as a source of tissue-specific antigens that are taken up by DCs and presented in the absence of co-stimulation, leading to T-cell tolerance [32]. DCs are well-equipped to sense the presence of danger signals from pathogens, known as pathogen-associated molecular pattern (PAMP) molecules, and from stressed or damaged cells, known as DAMPs [33]. DCs that are exposed to PAMPs and/or DAMPs upregulate the expression of co-stimulatory molecules. Thus, in the presence of an infection or other inflammatory condition that generates PAMPs and DAMPs, self-antigens can be presented by DCs that express costimulatory molecules. Because of their critical contribution to the activation of the immune response, these danger signals are known as ‘adjuvants’. Nevertheless, T cells do not usually react to self-antigens, at least in part because only T cells with TCRs that have low affinity for self-peptide–MHC complexes graduate thymic education. By contrast, peptides derived from foreign proteins, such as those encoded by viruses, are recognized by high-affinity TCRs.

The exquisite specificity of CD8 T-cell-mediated responses for infected cells is not, however, just a matter of antigen quality. Elegant studies investigating the quantitative aspects of antigen processing and presentation by MHC-I have revealed that the likelihood that a peptide generated by the proteasome will be presented by MHC-I is also a numbers game (reviewed in [34, 35]). Only a small fraction of the peptides generated by the proteasome, estimated at < 0.1%, is presented by MHC-I molecules [27]. In order to secure efficient and timely presentation of viral antigens during an acute infection, the system is skewed towards newly synthesized proteins, which are the main source of peptides presented by MHC-I. In other words, the rate of synthesis of an antigen is more important than the amount of antigen present in the cell for its recognition by T cells [34, 36].

Finally, while all cells express the standard proteasome, DCs constitutively express high levels of the immunoproteasome, a specialized variant that differs from the standard proteasome in three subunits and that cleaves slightly differently, generating peptides that are more suitable for MHC-I binding [37]. In normal conditions, expression of the immunoproteasome is very low in non-immune cells, but it is enhanced in inflammatory conditions by several cytokines, including interferon (IFN) type I (IFN-I) and type II (IFN-II). During a viral infection, activation of the IFN-I and NF-κB pathways provides signals for the recruitment and maturation of DCs to take up viral antigens from dying infected cells and cross-present them to CD8 T cells. The same pathways fine-tune the antigen presentation machinery of the infected cells to generate and present the same antigenic peptides towards which the T cells have been activated by DCs [38]. As discussed below, the ability of radiotherapy to enhance tumor immunogenicity is likely to depend on the induction of a state of viral mimicry in the cancer cells.

## How radiation modulates antigen presentation by cancer cells

A bulk of work in pre-clinical tumor models, supported by clinical observations, provided the rationale for the hypothesis that focal tumor RT can convert the tumor into an in situ, individualized vaccine [39]. Irradiated cancer cells undergo a stressful death that is associated with the release of DAMPs, such as the high-mobility-group Box 1 (HMGB1) alarmin protein [40], and the upregulation of signals that promote their phagocytosis by DCs, such as calreticulin [41]. This fate is shared by cancer cells that are treated with chemotherapy agents such as anthracyclines and oxaliplatin [42]. In addition, our recent studies have revealed a key role of radiation-induced viral mimicry in the stimulation of robust tumor-specific CD8 T-cell responses that are capable of mediating systemic tumor regression in concert with ICB therapy [25, 43].

IFN-I plays a central role in anti-viral immune responses. Its activation is triggered by the accumulation of viral DNA in the cytosolic compartment of infected cells. Cytosolic DNA is sensed by the cyclic GMP-AMP synthase (cGAS) [44]. cGAS catalyzes the formation of the cyclic dinucleotide cGAMP (cyclic guanosine monophosphate–adenosine monophosphate), which binds to transmembrane protein 173 (TMEM173, also known as stimulator of IFN genes (STING)). STING recruits the TANK-binding kinase 1 (TBK1), which phosphorylates interferon regulatory factor 3 (IRF3), enabling IFN-I gene transcription. IκB kinase (IKK), which phosphorylates IκB, is also recruited, resulting in IκB proteosomal degradation and canonical NF-κB signaling [45]. Radiation induces DNA breaks that trigger the DNA damage response. During this process, self-DNA accumulates in the cytosol of cancer cells at detectable levels, leading to activation of the cGAS–STING pathway and the resultant production of the type I interferon IFN-β by the irradiated cancer cells, which is comparable to the production of IFN-I that is observed upon viral infection of the same cells [43]. The relative contributions of genomic and mitochondrial DNA to the IFN-stimulatory cytosolic DNA in irradiated cells remain to be determined. Micronuclei, which are cytoplasmic aggregates of damaged DNA encircled by a defective nuclear envelope, have been shown to form following RT-induced DNA damage and to be major contributors to the pool of DNA that stimulates cGAS [46, 47].

The burst in IFN-I production by cancer cells following RT promotes the recruitment of DCs that are specialized in the cross-presentation of tumor antigens to CD8 T cells. In the setting of the release of DAMPs by cancer cells in an irradiated tumor microenvironment, these DCs upregulated costimulatory molecules and activated tumor-specific CD8 T cells [43]. Tumor-derived DNA itself has also been shown to be a DAMP that stimulates the cGAS–STING pathway in DCs, inducing them to produce IFN-I [48]. It remains unclear whether the tumor-cell DNA reaches the cytosol of DCs during phagocytosis, a process that is limited by CD47–SIRPα (signal regulatory protein α) interaction [49], or via other mechanisms. A possible mechanism by which tumor-cell DNA might reach the cytosol of DCs is shuttling by exosomes that are secreted by irradiated cancer cells, which have been shown to transfer IFN-stimulatory DNA to DCs in vitro, but the role of this mechanism in vivo remains to be fully elucidated [50].

The viral mimicry of radiation is not limited to the production of DAMPs and the activation of pro-inflammatory cytokines. Radiation modulates the expression of a large number of genes, many of which are involved in DNA repair [51]. As described above, newly synthesized proteins are the preferred source of peptides for MHC-I presentation. Thus, similar to proteins that are derived from viral antigens during an acute infection, the proteome that is acutely induced in response to ionizing radiation is the source of the antigens presented by irradiated cancer cells. Evidence in support of this hypothesis comes from studies by Reits and colleagues [52], who characterized the peptides presented by MHC-I of irradiated and non-irradiated melanoma cells (of the MelJuSo cell line) by mass spectrometry and identified several peptides that are unique to the irradiated cells. Among them were peptides derived from proteins that are involved in DNA repair and in protein breakdown. Additional evidence comes from our analysis of a non-small cell lung cancer (NSCLC) patient treated with RT and the ICB therapy ipilimumab [25].

It is also important to consider that different radiation doses and delivery schedules will induce the expression of different sets of genes [53]. We have shown that multi-dose radiation regimens (8 Gray (Gy) given on three consecutive days (8GyX3); and 6GyX5) induced systemic anti-tumor immune responses in combination with ICB, whereas a single dose of 20 Gy did not [17]. In-depth mechanistic studies revealed that single doses in excess of 10–15 Gy, depending on the cancer cells studied, did not induce an IFN-I response because the cytosolic DNA was cleared by the exonuclease TREX1 [43]. Consequently, a large set of IFN-stimulated genes was upregulated in cancer cells treated with 8GyX3 but not in those treated with 20GyX1. These findings suggest that the proteome presented by MHC-I on cancer cells, and on the cross-presenting DCs that take up the tumor antigens after radiation exposure, may vary significantly depending on the dose per fraction of radiation applied. Moreover, given the role of IFN-I in enhancing the expression of the immunoproteasome [54], it can be hypothesized that the repertoire of antigens presented by irradiated cancer cells is likely to be fine-tuned to match the repertoire presented by DCs only following RT doses that optimally stimulate the cGAS–STING pathway [55].

Overall, preclinical and clinical evidence suggests that RT, in addition to the recruitment of DCs specialized in cross-presentation of tumor antigens to CD8 T cells, can enhance tumor antigenicity by inducing a ‘burst’ of gene transcription that is likely to provide many new and potentially immunogenic peptides for loading onto MHC-I of both cross-presenting DCs and cancer cells.

## Radiation and the cancer mutanome

Ionizing radiation and DNA-damaging chemotherapy are powerful mutagens: cancer cells that survive these treatments often carry new mutations. Ionizing-radiation-induced mutagenesis is a stochastic cell-specific process, and it is generally considered highly unlikely that the same mutation will be generated in more than one cell following irradiation [56]. The ability of the immune system to reject a tumor depends on the proportion of cancer cells that present an antigen [57]. Like cytotoxic chemotherapy (for example, using alkylating agents), subclonal mutations induced by radiation may increase the mutational load without increasing the sensitivity of the tumor to ICB therapy [57], suggesting that they do not constitute good targets for tumor rejection. It is worth mentioning, however, that radiation-induced immunogenic variants could theoretically serve as important antigens in radiation-induced secondary cancers, or when treating a relapsed tumor in which evolutionary pressure selected for cells carrying the radiation-induced mutation.

As discussed earlier, the radiation-induced proteome is presented by MHC-I of irradiated cancer cells (Fig. 1). This implies that, in response to radiation, the expression of genes encoding proteins that are involved in cellular stress and DNA damage repair is induced. Furthermore, as these genes might contain mutations, at least some of these otherwise silent immunogenic mutations could be exposed to the immune system. This process could represent an important mechanism whereby RT enhances responses to ICB in patients who have cancers with a high mutational burden, such as melanoma and NSCLC [23, 24]. Further support for this notion comes from our recent study of metastatic NSCLC patients who were enrolled in a prospective trial of RT and ipilimumab. Objective abscopal responses were observed in 18% of these patients. In-depth functional analysis in one patient, who had a complete response, revealed a rapid in vivo expansion of CD8 T cells recognizing a neoantigen encoded by the *KPNA2* gene, a radiation-upregulated gene [25, 58].

It remains to be determined how often these otherwise silent immunogenic mutations are expressed and presented by MHC-I in irradiated cancer cells. More than 150 different molecules are involved in DNA repair alone, a process that is highly dependent on the cell cycle [59]. Radiation induces the expression of genes encoding proteins that are involved in DNA-repair mechanisms and those encoding multiple cell-cycle regulators. These same genes are frequently mutated in cancer cells, resulting in uncontrolled proliferation and genomic instability [60]. It follows that the molecules that are upregulated in irradiated cancer cells are encoded by a set of genes that are rich in mutations, increasing the likelihood that some of them will be immunogenic. We are currently analyzing multiple tumors and performing mass spectrometry of MHC-I-bound peptides to assess the differences in presented antigens between untreated and irradiated cancer cells.

## ‘Spreading the news’: the role of MHC-II in presenting the cancer mutanome

The anti-tumor immune response against neoantigens that are expressed by solid tumors is predominantly attributed to MHC-I-restricted CD8 cytotoxic T cells, but MHC-II-restricted CD4 T cells are also important drivers of anti-tumor immunity [61–63]. With some exceptions, MHC-II molecules are not expressed by solid tumors, but are selectively expressed by antigen-presenting cells (APCs), including DCs, B cells, and macrophages. Therefore, CD4 T cells do not directly target cancer cells but promote the cross-priming of CD8 T cells to tumor antigens by CD40 ligand-mediated DC activation [64].

MHC-II molecules present peptides that are derived from a large variety of endogenous and exogenous proteins that are degraded in the endosomal pathway [65]. The MHC-II antigen processing and presentation pathways vary depending on the type of APC; this complexity has been extensively reviewed [28, 66] and is not addressed here. Of note is the fact that, in mouse solid tumor models, a larger portion of the immunogenic mutanome was presented by MHC-II than MHC-I, and was recognized by CD4 T cells, possibly because of the less stringent length and sequence requirements for peptide binding to MHC-II than MHC-I molecules [61]. The vaccination of mice bearing established CT26 colorectal tumors that had multiple MHC-II-restricted neoepitopes elicited tumor regression, which was mediated by CD8 T cells that recognized a non-mutated neoepitope encoded by an endogenous retrovirus [61]. This evidence emphasizes the critical role of CD4 T cells in promoting the cross-priming of tumor-specific CD8 T cells [61]. Mutations in genes encoding peptides that are predicted to bind to MHC-II were also found to be abundant in human cancers, although their role in response to ICB remains to be determined [61]. Further supporting the importance of neoantigen-specific CD4 T-cell responses, in a personalized vaccine trial in melanoma patients, polyfunctional CD4 T cells were observed against 60% of the 97 unique neoantigens used across patients, whereas only 16% were recognized by CD8 T cells [8].

As described above, CD4 T-cell responses that are specific for neoantigens exert their helper function at the level of the DC and enhance the activation of anti-tumor CD8 T cells [61]. The abundance of an antigen is critical to achieving an efficient presentation via the endosomal pathway of APCs [67], so it can be predicted that the radiation-induced mutanome may boost neoantigen presentation by MHC-II, enhancing the activation of CD4 T-helper responses. Moreover, radiation and chemotherapy have been shown to markedly enhance antigen transfer from cancer cells to myeloid cells that are present in the tumor stroma [68], thus spreading the news about the antigenic composition of the cancer cells, with potential consequences for T-cell priming and T-cell-mediated restructuring of the tumor microenvironment.

## Beyond the mutanome: the emerging role of other types of cancer neoantigen

Cancer neoantigens encoded by genes containing non-synonymous mutations have been the focus of most studies, but other types of cancer neoantigens are beginning to emerge as important targets of tumor-specific T cells. These include neoantigens generated by PTMs, proteasome splicing, or RNA splicing, or from non-coding regions of the DNA.

PTMs of proteins can give rise to peptides presented by MHC molecules that activate T-cell responses [27]. Examples of PTMs that are presented by MHC-I include phosphorylated and glycosylated peptides [69–71], but many other modifications (such as oxidation and hydrolysis) have been shown to alter the immunogenicity of MHC-I peptides [27]. There is evidence that phosphorylated peptides are recognized by tumor-specific T cells across different malignancies, suggesting that they could represent shared antigens that are associated with altered phosphorylation pathways in tumors [71, 72]. Likewise, MHC-II molecules present modified peptides, and many of these modifications have been linked to allergic and autoimmune diseases [27]. MHC-II-restricted phosphopeptides have also been reported as relevant targets for human CD4 T cells [73]. In addition, a recent study showed that self-antigens that are modified by citrullination on tumor cells can mediate potent anti-tumor CD4 T-cell responses [74].

Peptide splicing by the standard proteasome is another mechanism that increases the diversity of the antigenic peptides presented to CD8 T cells [75, 76]. Liepe et al. [77] reported that proteasome-generated spliced peptides accounted for about one third of the MHC-I immunopeptidome in terms of diversity and one quarter in terms of abundance. To our knowledge, no MHC-II-restricted neoepitopes generated by proteasome splicing have been reported in tumors, but a study demonstrated that autoreactive CD4 T cells in type I diabetes recognize MHC-II epitopes formed by peptide fusion in β cells [78]. Thus, it is possible that such processes could also occur in tumors.

In addition, non-coding DNA regions have been recently demonstrated to be a source of targetable tumor-specific antigens [79]. These so-called ‘cryptic’ MHC-associated peptides can be produced by translation of protein-coding genes in non-canonical reading frames or by translation of non-coding sequences. Finally, recent work has shown that tumor cells have up to 30% more alternative RNA splicing events than normal cells [80], although further studies are needed to determine whether these events lead to the generation of neoantigens that are recognized by T cells.

It remains to be determined whether treatment modulates the expression of these different types of tumor neoantigens. Some types of PTM, such as oxidation, are expected to be induced by RT and may generate another group of RT-specific neoantigens. Epigenetic modulators (DNA methyltransferase and histone deacetylase inhibitors) induce the transcription of cryptic genes, including the reactivation of endogenous retroviruses, leading to increased tumor immunogenicity [81, 82]. The impact of chemotherapy on alternative transcription and splicing has been reviewed extensively [83]. Small molecules are being screened for their utility as alternative splicing modulators (for example, digoxin), although their effects in combination with immunotherapy have not yet been evaluated [84].

## Conclusions and future directions

The field of cancer vaccines has struggled for a long time to identify shared tumor antigens that could be used to induce effective anti-tumor immune responses in patients [85]. Progress in genomic and proteomic analysis has enabled the identification of unique mutations and PTMs that are immunogenic and can elicit powerful anti-tumor T-cell responses. In developing strategies to enhance such tumor-specific T-cell responses, it is important to consider the complex biology of antigen presentation. Multiple combination treatments, including chemotherapy, RT, and epigenetic therapy, are being tested in combination with ICB. Each of these treatments can modulate the expression and MHC-presentation of the various categories of neoantigens.

We have discussed the evidence in support of the concept that RT-induced viral mimicry is not limited to the production of IFN-I, which promotes the recruitment and activation of DCs that are essential for the cross-presentation of tumor antigens to CD8 T cells [43, 86]. In addition, this mimicry extends to directing the T-cell response towards antigens derived from the radiation-induced proteome, similar to the preferential presentation of newly synthesized viral proteins upon acute infection [34, 36, 52]. Our recent data from an NSCLC patient responding to RT and ipilimumab provide supportive evidence that RT can enhance the expression of an immunogenic mutation in the irradiated tumor and can lead to priming of neoantigen-specific CD8 T cells [25].

Several questions remain to be answered, among them whether RT enhances the expression of the immunoproteasome in cancer cells, and how CD8 T cells that are specific for a radiation-exposed neoantigen manage to recognize and eliminate metastases outside the radiation field (that is, induce an abscopal response), where the neoantigen is expressed at lower levels [25]. It can be reasoned that the expression levels of an antigen are critical for the activation of naïve T cells by cross-presenting DCs, but once activated, effector CD8 T cells can target cancer cells that have lower antigen expression. It has also been shown that once a robust anti-tumor T-cell response is activated and cytotoxic T cells are driven into the tumor, they will promote antigen spread, that is, the broadening of the T-cell response to additional tumor antigens [87]. The latter mechanism may be crucial for the therapeutic success of all forms of intratumoral immunotherapy, which usually treat only one or a few accessible sites but can, in some cases, induce the regression of untreated metastases [88]. Evidence of TCR repertoire diversification in mouse tumors is consistent with the hypothesis that antigen spread occurs after RT and ICB [89, 90]. We are currently testing which of the expanded T-cell clones that are present in irradiated tumors home to abscopal tumor sites. The regression of some but not all metastases in some patients with metastatic disease who were treated with RT of a single lesion and ICB also suggests that tumor heterogeneity may be a barrier when insufficient antigenic overlap occurs between the irradiated tumor and non-irradiated metastases [25]. The irradiation of multiple metastases has been suggested as a strategy to overcome tumor heterogeneity [91]. Finally, in the setting of vaccination with neoantigens or adoptive T-cell therapy, RT could be used to facilitate the recognition and elimination of cancer cells if the neoantigen(s) that are targeted are upregulated by RT.

Despite the many open questions that are being addressed experimentally, we suggest that the expression of immunogenic mutations in genes that are modulated by radiation could be a candidate biomarker for predicting which tumors may benefit the most from RT, to enhance responses to ICB. The potential of RT to modulate antigenic mutations could be included in a comprehensive model aimed at understanding the determinants of responses to RT and ICB in the clinic. Other components of this model include the expression of molecules that are mechanistically linked to the priming of tumor-specific T cells by RT, such as cGAS and STING [55], and the expression of molecules that are linked to cancer-cell recognition by CD8 T cells, such as MHC-I, β2m, and components of the antigen-presentation machinery [92, 93]. Overall, the availability of new tools that allow in-depth analyses of the antigenic repertoire of cancer cells and the immune responses that they engender opens new opportunities to design rational treatment combinations to improve patients’ responses.

Box 1 Glossary **Abscopal effect:** this indicates tumor regression that occurs outside the field of radiation.

**Adjuvant:** a substance or molecule that enhances the immune response to an antigen, usually by activating innate immune cells. Adjuvants can be derived from pathogens or from stressed cells, in which case they are considered ‘endogenous’ adjuvants.

**Cross-presentation:** the ability of some antigen-presenting cells to take up and present exogenous antigens with MHC class I molecules to CD8 T cells, via the route normally employed for endogenous antigens.

**Cyclic GMP-AMP synthase (cGAS):** an enzyme that catalyzes cyclic GMP-AMP synthesis and acts as a cytosolic DNA sensor that binds to microbial DNA as well as to self-DNA.

**Damage-associated molecular patterns (DAMPs):** endogenous molecules that operate as endogenous adjuvants when released by stressed or dying cells.

**Differentiation antigen:** an antigen derived from a protein that is expressed in a specific tissue or organ and the tumor derived from this tissue.

**Immune checkpoint blockade (ICB):** a therapeutic strategy based on the inhibition of immune checkpoint receptors expressed by T cells that are in place to maintain self-tolerance and are co-opted by cancers to evade immune rejection.

**Micronuclei:** extranuclear bodies that contain damaged chromosome fragments that are not incorporated into the nucleus after cell division.

**Pathogen-associated molecular patterns (PAMPs):** conserved molecular motifs that are expressed by pathogens and recognized by receptors of the innate immune system as signals of danger.

**Post-translational modification (PTM):** a biochemical modification of a protein that occurs after translation.

**Radiation dose:** the energy deposited by ionizing radiation per unit mass, measured in Gray (Gy): 1 Gy = 1 J/kg.

**Stimulator of interferon genes (STING):** an endoplasmic-reticulum-associated protein that activates the type I IFN and NF-κB pathways. STING is activated by cyclic GMP-AMP produced by cGAS and by other cyclic dinucleotides of bacterial origin.

## Abbreviations

APC: Antigen-presenting cell
cGAS: Cyclic GMP-AMP synthase
DAMP: Damage-associated molecular pattern
DC: Dendritic cell
ER: Endoplasmic reticulum
ICB: Immune checkpoint blockade
IFN: Interferon
IKK: IκB kinase
MHC-I: Major histocompatibility complex class I
NSCLC: Non-small cell lung cancer
PAMP: Pathogen-associated molecular pattern
PTM: Post-translational modification
RT: Radiotherapy
STING: Stimulator of interferon genes
TCR: T-cell receptor
β2m: β2-microglobulin

## References

[1] Scanlan MJ, Gure AO, Jungbluth AA, Old LJ, Chen YT (2002). Cancer/testis antigens: an expanding family of targets for cancer immunotherapy. Immunol Rev.

[2] Finn OJ (2017). Human tumor antigens yesterday, today, and tomorrow. Cancer Immunol Res..

[3] Snyder A, Makarov V, Merghoub T, Yuan J, Zaretsky JM, Desrichard A (2014). Genetic basis for clinical response to CTLA-4 blockade in melanoma. N Engl J Med.

[4] Rizvi NA, Hellmann MD, Snyder A, Kvistborg P, Makarov V, Havel JJ (2015). Cancer immunology. Mutational landscape determines sensitivity to PD-1 blockade in non-small cell lung cancer. Science..

[5] Giannakis M, Mu XJ, Shukla SA, Qian ZR, Cohen O, Nishihara R (2016). Genomic correlates of immune-cell infiltrates in colorectal carcinoma. Cell Rep.

[6] Le DT, Durham JN, Smith KN, Wang H, Bartlett BR, Aulakh LK (2017). Mismatch repair deficiency predicts response of solid tumors to PD-1 blockade. Science..

[7] Schumacher TN, Schreiber RD (2015). Neoantigens in cancer immunotherapy. Science..

[8] Ott PA, Hu Z, Keskin DB, Shukla SA, Sun J, Bozym DJ (2017). An immunogenic personal neoantigen vaccine for patients with melanoma. Nature..

[9] Keskin DB, Anandappa AJ, Sun J, Tirosh I, Mathewson ND, Li S (2019). Neoantigen vaccine generates intratumoral T cell responses in phase Ib glioblastoma trial. Nature..

[10] Sahin U, Derhovanessian E, Miller M, Kloke BP, Simon P, Lower M (2017). Personalized RNA mutanome vaccines mobilize poly-specific therapeutic immunity against cancer. Nature..

[11] Luksza M, Riaz N, Makarov V, Balachandran VP, Hellmann MD, Solovyov A (2017). A neoantigen fitness model predicts tumour response to checkpoint blockade immunotherapy. Nature..

[12] Balachandran VP, Luksza M, Zhao JN, Makarov V, Moral JA, Remark R (2017). Identification of unique neoantigen qualities in long-term survivors of pancreatic cancer. Nature..

[13] Boehm KM, Bhinder B, Raja VJ, Dephoure N, Elemento O (2019). Predicting peptide presentation by major histocompatibility complex class I: an improved machine learning approach to the immunopeptidome. BMC Bioinformatics.

[14] Rodriguez-Ruiz ME, Vanpouille-Box C, Melero I, Formenti SC, Demaria S (2018). Immunological mechanisms responsible for radiation-induced abscopal effect. Trends Immunol.

[15] Wilkins AC, Patin EC, Harrington KJ, Melcher AA (2019). The immunological consequences of radiation-induced DNA damage. J Pathol.

[16] Lugade AA, Moran JP, Gerber SA, Rose RC, Frelinger JG, Lord EM (2005). Local radiation therapy of B16 melanoma tumors increases the generation of tumor antigen-specific effector cells that traffic to the tumor. J Immunol.

[17] Dewan MZ, Galloway AE, Kawashima N, Dewyngaert JK, Babb JS, Formenti SC (2009). Fractionated but not single-dose radiotherapy induces an immune-mediated abscopal effect when combined with anti-CTLA-4 antibody. Clin Cancer Res.

[18] Lee Y, Auh SL, Wang Y, Burnette B, Wang Y, Meng Y (2009). Therapeutic effects of ablative radiation on local tumor require CD8+ T cells: changing strategies for cancer treatment. Blood..

[19] Schaue D, Comin-Anduix B, Ribas A, Zhang L, Goodglick L, Sayre JW (2008). T-cell responses to survivin in cancer patients undergoing radiation therapy. Clin Cancer Res.

[20] Formenti SC, Demaria S (2009). Systemic effects of local radiotherapy. Lancet Oncol.

[21] Abuodeh Y, Venkat P, Kim S (2016). Systematic review of case reports on the abscopal effect. Curr Probl Cancer.

[22] Demaria S, Kawashima N, Yang AM, Devitt ML, Babb JS, Allison JP (2005). Immune-mediated inhibition of metastases after treatment with local radiation and CTLA-4 blockade in a mouse model of breast cancer. Clin Cancer Res.

[23] Postow MA, Callahan MK, Barker CA, Yamada Y, Yuan J, Kitano S (2012). Immunologic correlates of the abscopal effect in a patient with melanoma. N Engl J Med.

[24] Golden EB, Demaria S, Schiff PB, Chachoua A, Formenti SC (2013). An abscopal response to radiation and ipilimumab in a patient with metastatic non-small cell lung cancer. Cancer Immunol Res.

[25] Formenti SC, Rudqvist NP, Golden E, Cooper B, Wennerberg E, Lhuillier C (2018). Radiotherapy induces responses of lung cancer to CTLA-4 blockade. Nat Med.

[26] Rock KL, Reits E, Neefjes J (2016). Present yourself! By MHC class I and MHC class II molecules. Trends Immunol.

[27] Neefjes J, Ovaa H (2013). A peptide's perspective on antigen presentation to the immune system. Nat Chem Biol.

[28] Neefjes J, Jongsma ML, Paul P, Bakke O (2011). Towards a systems understanding of MHC class I and MHC class II antigen presentation. Nat Rev Immunol..

[29] Banchereau J, Steinman RM (1998). Dendritic cells and the control of immunity. Nature..

[30] Peggs KS, Allison JP (2005). Co-stimulatory pathways in lymphocyte regulation: the immunoglobulin superfamily. Br J Haematol.

[31] Hildner K, Edelson BT, Purtha WE, Diamond M, Matsushita H, Kohyama M (2008). Batf3 deficiency reveals a critical role for CD8alpha+ dendritic cells in cytotoxic T cell immunity. Science..

[32] Steinman RM, Hawiger D, Liu K, Bonifaz L, Bonnyay D, Mahnke K (2003). Dendritic cell function in vivo during the steady state: a role in peripheral tolerance. Ann N Y Acad Sci.

[33] Pulendran B, Palucka K, Banchereau J (2001). Sensing pathogens and tuning immune responses. Science..

[34] Yewdell JW, Reits E, Neefjes J (2003). Making sense of mass destruction: quantitating MHC class I antigen presentation. Nat Rev Immunol..

[35] Anton LC, Yewdell JW (2014). Translating DRiPs: MHC class I immunosurveillance of pathogens and tumors. J Leukoc Biol.

[36] Khan S, de Giuli R, Schmidtke G, Bruns M, Buchmeier M, van den Broek M (2001). Cutting edge: neosynthesis is required for the presentation of a T cell epitope from a long-lived viral protein. J Immunol.

[37] Ferrington DA, Gregerson DS (2012). Immunoproteasomes: structure, function, and antigen presentation. Prog Mol Biol Transl Sci.

[38] Murata S, Takahama Y, Kasahara M, Tanaka K (2018). The immunoproteasome and thymoproteasome: functions, evolution and human disease. Nat Immunol.

[39] Formenti SC, Demaria S (2012). Radiotherapy to convert the tumor into an in situ vaccine. Int J Radiat Oncol Biol Phys.

[40] Apetoh L, Ghiringhelli F, Tesniere A, Obeid M, Ortiz C, Criollo A (2007). Toll-like receptor 4-dependent contribution of the immune system to anticancer chemotherapy and radiotherapy. Nat Med.

[41] Obeid M, Panaretakis T, Joza N, Tufi R, Tesniere A, van Endert P (2007). Calreticulin exposure is required for the immunogenicity of gamma-irradiation and UVC light-induced apoptosis. Cell Death Differ.

[42] Kroemer G, Galluzzi L, Kepp O, Zitvogel L (2013). Immunogenic cell death in cancer therapy. Annu Rev Immunol.

[43] Vanpouille-Box C, Alard A, Aryankalayil MJ, Sarfraz Y, Diamond JM, Schneider RJ, et al. DNA exonuclease Trex1 regulates radiotherapy-induced tumour immunogenicity. Nat Commun. 2017. 10.1038/ncomms15618. 10.1038/ncomms15618 PMC5472757 28598415

[44] Cai X, Chiu YH, Chen ZJ (2014). The cGAS-cGAMP-STING pathway of cytosolic DNA sensing and signaling. Mol Cell.

[45] Galluzzi L, Vanpouille-Box C, Bakhoum SF, Demaria S (2018). SnapShot: CGAS-STING signaling. Cell..

[46] Harding SM, Benci JL, Irianto J, Discher DE, Minn AJ, Greenberg RA (2017). Mitotic progression following DNA damage enables pattern recognition within micronuclei. Nature..

[47] Mackenzie KJ, Carroll P, Martin CA, Murina O, Fluteau A, Simpson DJ (2017). cGAS surveillance of micronuclei links genome instability to innate immunity. Nature..

[48] Deng L, Liang H, Xu M, Yang X, Burnette B, Arina A (2014). STING-dependent cytosolic DNA sensing promotes radiation-induced type I interferon-dependent antitumor immunity in immunogenic tumors. Immunity..

[49] Xu MM, Pu Y, Han D, Shi Y, Cao X, Liang H (2017). Dendritic cells but not macrophages sense tumor mitochondrial DNA for cross-priming through signal regulatory protein α signaling. Immunity..

[50] Diamond JM, Vanpouille-Box C, Spada S, Rudqvist NP, Chapman JR, Ueberheide BM (2018). Exosomes shuttle TREX1-sensitive IFN-stimulatory dsDNA from irradiated cancer cells to DCs. Cancer Immunol Res..

[51] Amundson SA, Bittner M, Fornace AJ (2003). Functional genomics as a window on radiation stress signaling. Oncogene..

[52] Reits EA, Hodge JW, Herberts CA, Groothuis TA, Chakraborty M, Wansley EK (2006). Radiation modulates the peptide repertoire, enhances MHC class I expression, and induces successful antitumor immunotherapy. J Exp Med.

[53] Tsai MH, Cook JA, Chandramouli GV, DeGraff W, Yan H, Zhao S (2007). Gene expression profiling of breast, prostate, and glioma cells following single versus fractionated doses of radiation. Cancer Res.

[54] Shin EC, Seifert U, Kato T, Rice CM, Feinstone SM, Kloetzel PM (2006). Virus-induced type I IFN stimulates generation of immunoproteasomes at the site of infection. J Clin Invest.

[55] Vanpouille-Box C, Formenti SC, Demaria S (2018). Toward precision radiotherapy for use with immune checkpoint blockers. Clin Cancer Res.

[56] Grosovsky AJ, de Boer JG, de Jong PJ, Drobetsky EA, Glickman BW (1988). Base substitutions, frameshifts, and small deletions constitute ionizing radiation-induced point mutations in mammalian cells. Proc Natl Acad Sci U S A.

[57] McGranahan N, Furness AJ, Rosenthal R, Ramskov S, Lyngaa R, Saini SK (2016). Clonal neoantigens elicit T cell immunoreactivity and sensitivity to immune checkpoint blockade. Science..

[58] Song KH, Jung SY, Kang SM, Kim MH, Ahn J, Hwang SG (2016). Induction of immunogenic cell death by radiation-upregulated karyopherin alpha 2 in vitro. Eur J Cell Biol.

[59] Wood RD, Mitchell M, Lindahl T (2005). Human DNA repair genes, 2005. Mutat Res.

[60] Jeggo PA, Pearl LH, Carr AM (2016). DNA repair, genome stability and cancer: a historical perspective. Nat Rev Cancer.

[61] Kreiter S, Vormehr M, van de Roemer N, Diken M, Lower M, Diekmann J (2015). Mutant MHC class II epitopes drive therapeutic immune responses to cancer. Nature..

[62] Linnemann C, van Buuren MM, Bies L, Verdegaal EM, Schotte R, Calis JJ (2015). High-throughput epitope discovery reveals frequent recognition of neo-antigens by CD4+ T cells in human melanoma. Nat Med.

[63] Tran E, Turcotte S, Gros A, Robbins PF, Lu YC, Dudley ME (2014). Cancer immunotherapy based on mutation-specific CD4+ T cells in a patient with epithelial cancer. Science..

[64] Schoenberger SP, Toes RE, van der Voort EI, Offringa R, Melief CJ (1998). T-cell help for cytotoxic T lymphocytes is mediated by CD40–CD40L interactions. Nature..

[65] Stern LJ, Santambrogio L (2016). The melting pot of the MHC II peptidome. Curr Opin Immunol.

[66] Roche PA, Furuta K (2015). The ins and outs of MHC class II-mediated antigen processing and presentation. Nat Rev Immunol.

[67] Shen Z, Reznikoff G, Dranoff G, Rock KL (1997). Cloned dendritic cells can present exogenous antigens on both MHC class I and class II molecules. J Immunol.

[68] Zhang B, Bowerman NA, Salama JK, Schmidt H, Spiotto MT, Schietinger A (2007). Induced sensitization of tumor stroma leads to eradication of established cancer by T cells. J Exp Med.

[69] Andersen MH, Bonfill JE, Neisig A, Arsequell G, Sondergaard I, Valencia G (1999). Phosphorylated peptides can be transported by TAP molecules, presented by class I MHC molecules, and recognized by phosphopeptide-specific CTL. J Immunol.

[70] Haurum JS, Hoier IB, Arsequell G, Neisig A, Valencia G, Zeuthen J (1999). Presentation of cytosolic glycosylated peptides by human class I major histocompatibility complex molecules in vivo. J Exp Med.

[71] Cobbold M., De La Pena H., Norris A., Polefrone J. M., Qian J., English A. M., Cummings K. L., Penny S., Turner J. E., Cottine J., Abelin J. G., Malaker S. A., Zarling A. L., Huang H.-W., Goodyear O., Freeman S. D., Shabanowitz J., Pratt G., Craddock C., Williams M. E., Hunt D. F., Engelhard V. H. (2013). MHC Class I-Associated Phosphopeptides Are the Targets of Memory-like Immunity in Leukemia. Science Translational Medicine.

[72] Zarling AL, Polefrone JM, Evans AM, Mikesh LM, Shabanowitz J, Lewis ST (2006). Identification of class I MHC-associated phosphopeptides as targets for cancer immunotherapy. Proc Natl Acad Sci U S A.

[73] Depontieu FR, Qian J, Zarling AL, McMiller TL, Salay TM, Norris A (2009). Identification of tumor-associated, MHC class II-restricted phosphopeptides as targets for immunotherapy. Proc Natl Acad Sci U S A.

[74] Brentville VA, Metheringham RL, Gunn B, Symonds P, Daniels I, Gijon M (2016). Citrullinated vimentin presented on MHC-II in tumor cells is a target for CD4+ T-cell-mediated antitumor immunity. Cancer Res.

[75] Vigneron N, Stroobant V, Chapiro J, Ooms A, Degiovanni G, Morel S (2004). An antigenic peptide produced by peptide splicing in the proteasome. Science..

[76] Dalet A, Robbins PF, Stroobant V, Vigneron N, Li YF, El-Gamil M (2011). An antigenic peptide produced by reverse splicing and double asparagine deamidation. Proc Natl Acad Sci U S A.

[77] Liepe J, Marino F, Sidney J, Jeko A, Bunting DE, Sette A (2016). A large fraction of HLA class I ligands are proteasome-generated spliced peptides. Science..

[78] Delong T, Wiles TA, Baker RL, Bradley B, Barbour G, Reisdorph R (2016). Pathogenic CD4 T cells in type 1 diabetes recognize epitopes formed by peptide fusion. Science..

[79] Laumont Céline M., Vincent Krystel, Hesnard Leslie, Audemard Éric, Bonneil Éric, Laverdure Jean-Philippe, Gendron Patrick, Courcelles Mathieu, Hardy Marie-Pierre, Côté Caroline, Durette Chantal, St-Pierre Charles, Benhammadi Mohamed, Lanoix Joël, Vobecky Suzanne, Haddad Elie, Lemieux Sébastien, Thibault Pierre, Perreault Claude (2018). Noncoding regions are the main source of targetable tumor-specific antigens. Science Translational Medicine.

[80] Kahles A, Lehmann KV, Toussaint NC, Huser M, Stark SG, Sachsenberg T (2018). Comprehensive analysis of alternative splicing across tumors from 8,705 patients. Cancer Cell.

[81] Brocks D, Schmidt CR, Daskalakis M, Jang HS, Shah NM, Li D (2017). DNMT and HDAC inhibitors induce cryptic transcription start sites encoded in long terminal repeats. Nat Genet.

[82] Jones PA, Ohtani H, Chakravarthy A, De Carvalho DD (2019). Epigenetic therapy in immune-oncology. Nat Rev Cancer.

[83] Lambert CA, Garbacki N, Colige AC (2017). Chemotherapy induces alternative transcription and splicing: facts and hopes for cancer treatment. Int J Biochem Cell Biol.

[84] Stoilov P, Lin CH, Damoiseaux R, Nikolic J, Black DL (2008). A high-throughput screening strategy identifies cardiotonic steroids as alternative splicing modulators. Proc Natl Acad Sci U S A.

[85] Melief CJ, van Hall T, Arens R, Ossendorp F, van der Burg SH (2015). Therapeutic cancer vaccines. J Clin Invest.

[86] Hammerich L, Marron TU, Upadhyay R, Svensson-Arvelund J, Dhainaut M, Hussein S (2019). Systemic clinical tumor regressions and potentiation of PD1 blockade with in situ vaccination. Nat Med.

[87] Gulley JL, Madan RA, Pachynski R, Mulders P, Sheikh NA, Trager J, et al. Role of antigen spread and distinctive characteristics of immunotherapy in cancer treatment. J Natl Cancer Inst. 2017. 10.1093/jnci/djw261. 10.1093/jnci/djw261 PMC5441294 28376158

[88] Hammerich L, Bhardwaj N, Kohrt HE, Brody JD (2016). In situ vaccination for the treatment of cancer. Immunotherapy..

[89] Rudqvist NP, Pilones KA, Lhuillier C, Wennerberg E, Sidhom JW, Emerson RO (2018). Radiotherapy and CTLA-4 blockade shape the TCR repertoire of tumor-infiltrating T cells. Cancer Immunol Res..

[90] Twyman-Saint Victor C, Rech AJ, Maity A, Rengan R, Pauken KE, Stelekati E (2015). Radiation and dual checkpoint blockade activate non-redundant immune mechanisms in cancer. Nature..

[91] Brooks ED, Chang JY (2019). Time to abandon single-site irradiation for inducing abscopal effects. Nat Rev Clin Oncol.

[92] Garrido F, Algarra I (2001). MHC antigens and tumor escape from immune surveillance. Adv Cancer Res.

[93] Sade-Feldman M, Jiao YJ, Chen JH, Rooney MS, Barzily-Rokni M, Eliane JP, et al. Resistance to checkpoint blockade therapy through inactivation of antigen presentation. Nat Commun. 2017. 10.1038/s41467-017-01062-w. 10.1038/s41467-017-01062-w PMC5656607 29070816

